# Beyond psychiatry placements: advancing universal mental health competency across foundation training

**DOI:** 10.1192/bjb.2025.10143

**Published:** 2025-10

**Authors:** Veno Dwi Krisnanda

**Affiliations:** State University of Malang, Malang, Indonesia

The article in the recent issue of *BJPsych Bulletin* titled ‘The new Foundation Programme Mental Health Curriculum: foundation doctors’ perceptions of its importance and their competency: pre–post psychiatric placement evaluation study’ by Varvari et al^1^ provides insights regarding how psychiatric placements can significantly boost foundation doctors’ confidence in recognising and treating mental illnesses. In response to a global imperative, mental health has been recognised as a leading cause of disability-adjusted life years lost, yet its incorporation into early medical education remains fragmented.^2^ Future work should aim to expand on the findings and broaden the scope of the study of Varvari et al by not only confirming the benefits of placements but also questioning their viability as a fundamental method of mental health education. It will be vital to assess whether current models sufficiently and sustainably equip all physicians for the realities of modern clinical care, in which mental comorbidity is common and transcends settings.^2,3^

The quandary becomes more acute in the context of a more complex healthcare environment.^4^ Mental health symptoms are no longer restricted to mental health services; patients with these symptoms can also be seen in emergency departments, primary care clinics, post-COVID rehabilitation centres and long-term illness therapy settings.^4^ Our observations in clinical teaching contexts other than psychiatry reveal that foundation doctors have diverse capacities to assess psychological distress and navigate ethical–legal frameworks such as the Mental Capacity Act. Although direct exposure to psychiatric rotations is important, this is insufficient to ensure broad, transferable skills, particularly for practitioners who have never completed a mental health rotation.^5,6^ Access fragmentation causes disparities in both learning outcomes and patient care quality.^7–9^

This work offers an important addition to proving learning improvements through placement, especially in less common domains such as somatisation and personality disorders.^9^ However, a more nuanced interpretation of the findings suggests that the perceived relevance of scenario-based competencies, risk assessment, ethical reasoning and legal execution decreased after placement. This indicates a mismatch between curricular content and delivery environment. Although some success has been made, the advances may be confined to specific areas of introduction rather than comprehensive, context-responsive treatment. The lack of longitudinal examination at a behavioural or outcome level reduces interpretability throughout real-world practice.^3,9^

Given these constraints, a larger curriculum plan is required.^1^ Psychiatric competencies should be incorporated longitudinally across specialties, reinforced through interdisciplinary cooperation and strengthened using scalable modalities such as simulation-based e-learning, integrated case reviews and reflective team-based sessions.^10^ These improvements could guarantee that all trainees, not only those working in psychiatry, receive organised and experiential training. The advantages are global: in systems in which psychiatric services are sparse, integrated training can expand mental healthcare capacity across disciplines while reducing demand on mental health experts.^10^

Finally, physician mental health competency should be regarded as the cornerstone of safe, ethical and comprehensive medical treatment, rather than a requirement for placement.^9,11^ The studies considered here are an important starting point, but a more comprehensive systemic redesign is required to properly accomplish their aim. Including psychiatric education throughout the training continuum, spanning locations, disciplines and modalities, would better equip the global healthcare workforce to address current and future mental health concerns.^3,6^
